# Assessment of the stability of intracranial aneurysms using a deep learning model based on computed tomography angiography

**DOI:** 10.1007/s11547-024-01939-z

**Published:** 2024-12-12

**Authors:** Lu Zeng, Li Wen, Yang Jing, Jing-xu Xu, Chen-cui Huang, Dong Zhang, Guang-xian Wang

**Affiliations:** 1 Department of Radiology, Banan Hospital, Chongqing Medical University, Chongqing, 401320 China; 2 Department of Radiology, Xinqiao Hospital, The Second Affiliated Hospital of Army Medical University, Chongqing, 400037 China; 3 Huiying Medical Technology (Beijing), Beijing, 100192 China; 4 Department of Research Collaboration, R&D Center, Beijing Deepwise and League of PHD Technology Co., Ltd, No. A2, Xisanhuan North Road, Haidian District, Beijing, 100080 China

**Keywords:** Intracranial aneurysms, Computed tomography angiography, Deep learning, Convolutional neural network, Stability

## Abstract

**Purpose:**

Assessment of the stability of intracranial aneurysms is important in the clinic but remains challenging. The aim of this study was to construct a deep learning model (DLM) to identify unstable aneurysms on computed tomography angiography (CTA) images.

**Methods:**

The clinical data of 1041 patients with 1227 aneurysms were retrospectively analyzed from August 2011 to May 2021. Patients with aneurysms were divided into unstable (ruptured, evolving and symptomatic aneurysms) and stable (fortuitous, nonevolving and asymptomatic aneurysms) groups and randomly divided into training (833 patients with 991 aneurysms) and internal validation (208 patients with 236 aneurysms) sets. One hundred and ninety-seven patients with 229 aneurysms from another hospital were included in the external validation set. Six models based on a convolutional neural network (CNN) or logistic regression were constructed on the basis of clinical, morphological and deep learning (DL) features. The area under the curve (AUC), accuracy, sensitivity and specificity were calculated to evaluate the discriminating ability of the models.

**Results:**

The AUCs of Models A (clinical), B (morphological) and C (DL features from the CTA image) in the external validation set were 0.5706, 0.9665 and 0.8453, respectively. The AUCs of Model D (clinical and DL features), Model E (clinical and morphological features) and Model F (clinical, morphological and DL features) in the external validation set were 0.8395, 0.9597 and 0.9696, respectively.

**Conclusions:**

The CNN-based DLM, which integrates clinical, morphological and DL features, outperforms other models in predicting IA stability. The DLM has the potential to assess IA stability and support clinical decision-making.

**Supplementary Information:**

The online version contains supplementary material available at 10.1007/s11547-024-01939-z.

## Introduction

Unruptured intracranial aneurysms (UIAs) have been detected in approximately 3% of adults worldwide, with 7% being detected in China [1, 2]. Over the past few decades, an increasing number of UIAs have been detected incidentally due to the increased use of noninvasive imaging, including computed tomography angiography (CTA) and magnetic resonance angiography (MRA). Compared with MRA, CTA is a widely available and cost-effective diagnostic technique that allows rapid diagnosis and remains the first-line imaging modality with high sensitivity and specificity [3, 4]. However, the diagnostic sensitivity for small aneurysms is rather low, even for experienced radiologists. For example, for aneurysms with a diameter ≤ 3 mm, the sensitivity ranges from 64 to 74.1% [5]. Hence, in recent years, several articles describing the use of artificial intelligence (AI) for CTA have been published. Notably, the integration of AI models has significantly shortened the detection time and increased the detection rate of aneurysms [3, 6–9].

However, the reduction in detection time and increase in detection rate are insufficient. Approximately 85% of cases of nontraumatic subarachnoid hemorrhage (SAH) are caused by ruptured intracranial aneurysms (RIAs) [10], but most UIAs do not rupture [11]. Moreover, treatment methods for UIAs are likely to carry the risk of procedure-related complications. However, some UIAs may rupture despite conservative management, leading to serious consequences. Aneurysm stability over time is the only parameter, though it is an insufficient one, on which we can base the decision to continue with conservative management vs. to propose treatment to a patient. Hence, accurate and prompt differentiation between unstable and stable aneurysms optimizes clinical decision-making and thus leads to better outcomes [12].

The assessment of aneurysm stability is complex and involves many factors. Some clinical features and morphological features of aneurysms, such as age, SAH history, smoking history, maximum size, shape and location [13–15], have been shown to be related to aneurysm instability. The natural course of UIA studies, ELAPSS score (earlier SAH, location, age > 60 years, population, size and shape) and treatment score are used to help clinicians make optimal decisions [14–17]. However, these studies are mainly based on the conventional logistic regression (LR) method, and the relationship between the abovementioned risk factors and aneurysm stability is complex. The use of such scores in clinical settings is limited because of their low sensitivity [18–21]. In addition, there are several limitations of conventional LR, such as the inability to solve nonlinear problems, the difficulty of addressing data imbalances and the difficulty of fitting true distributions to data, which can lead to low sensitivity. Therefore, assessing the stability of aneurysms remains difficult.

AI has been used to detect and predict the rupture status of aneurysms. Machine learning (ML) is a branch of AI that outperforms conventional LR in terms of assessing nonlinear relationships and complex patterns [22]. Some ML models have been shown to be superior to traditional LR models [23, 24]. However, a recent study showed that ML models do not outperform conventional LR in predicting the rupture status of UIAs [25]. Deep learning (DL) is a subset of ML that uses multilayer neural networks (such as CNNs) to automatically learn features from data. DL models (DLMs) usually require large amounts of data for training but are able to automatically learn more complex features and achieve higher performance. For aneurysm research, DL is often used for automatic detection and segmentation of aneurysms on the basis of CTA images [3, 6–9], and ML is often used to predict aneurysm rupture risk assessment [23, 25–28]. However, few studies have focused on the use of DLMs for distinguishing unstable aneurysms from stable aneurysms. Hence, the purpose of this study was to construct a DLM that can distinguish unstable aneurysms on CTA images.

## Methods

### Patients

The study was approved by the Institutional Review Board at Banan Hospital and Xinqiao Hospital. The requirement for individual informed consent was waived because of the retrospective nature of the study, and the data were anonymized. The clinical data of patients with aneurysms who were consecutively admitted to our participating centers between August 2011 and May 2021 were retrospectively analyzed. The exclusion criteria for this study were as follows: (1) age younger than 18 years; (2) nonsaccular aneurysms, (such as fusiform, dissection, traumatic or infectious aneurysms); (3) diagnosed with vascular diseases (such as vascular malformations and moyamoya disease) and (4) incomplete clinical or CTA image data.

All the aneurysms were divided into two groups: stable aneurysms and unstable aneurysms. The criteria for unstable aneurysms included RIAs (more prone to rebleeding), aneurysm progression (size increase ≥ 1 mm, shape change or rupture) during follow-up on CTA or MRA and aneurysms associated with neurological symptoms (e.g., blepharoptosis) [16, 29, 30]. The remaining aneurysms were defined as stable aneurysms. Asymptomatic UIAs were followed for ≥ 3 months via CTA or MRA to determine whether the aneurysms were stable.

### Acquisition of clinical and morphological features

Patient clinical characteristics, including sex, age, alcohol consumption status, smoking status, hypertension status, diabetes mellitus status, cerebrovascular sclerosis status, heart disease status and history of aneurysmal SAH, were collected from medical records. Cerebrovascular sclerosis was defined as diffuse atherosclerosis of the brain, luminal stenosis and small vessel occlusion, the diagnosis was made on the basis of CTA or transcranial ultrasound.

All the CTA images were transferred to the GE Advantage workstation (Advantage Windows 4.5) to generate 3D-volume renderings (VRs). As in the previous studies, the morphological parameters of aneurysms were measured directly from 3D-VRs [31, 32]. All the CTA images were evaluated separately by two observers (one with 10 years of experience in vascular imaging and the other with 20 years of experience in neuroradiology) who were blinded to patient information and stability status. For patients with asymptomatic UIAs, follow-up images of the aneurysms were used to determine whether the aneurysm grew, and the first CTA study was used for analysis. Continuous data were calculated as average values obtained by the two observers. Discrepancies in categorical data were resolved by a third reader (with 25 years of experience in neuroradiology).

**Fig. 1:**
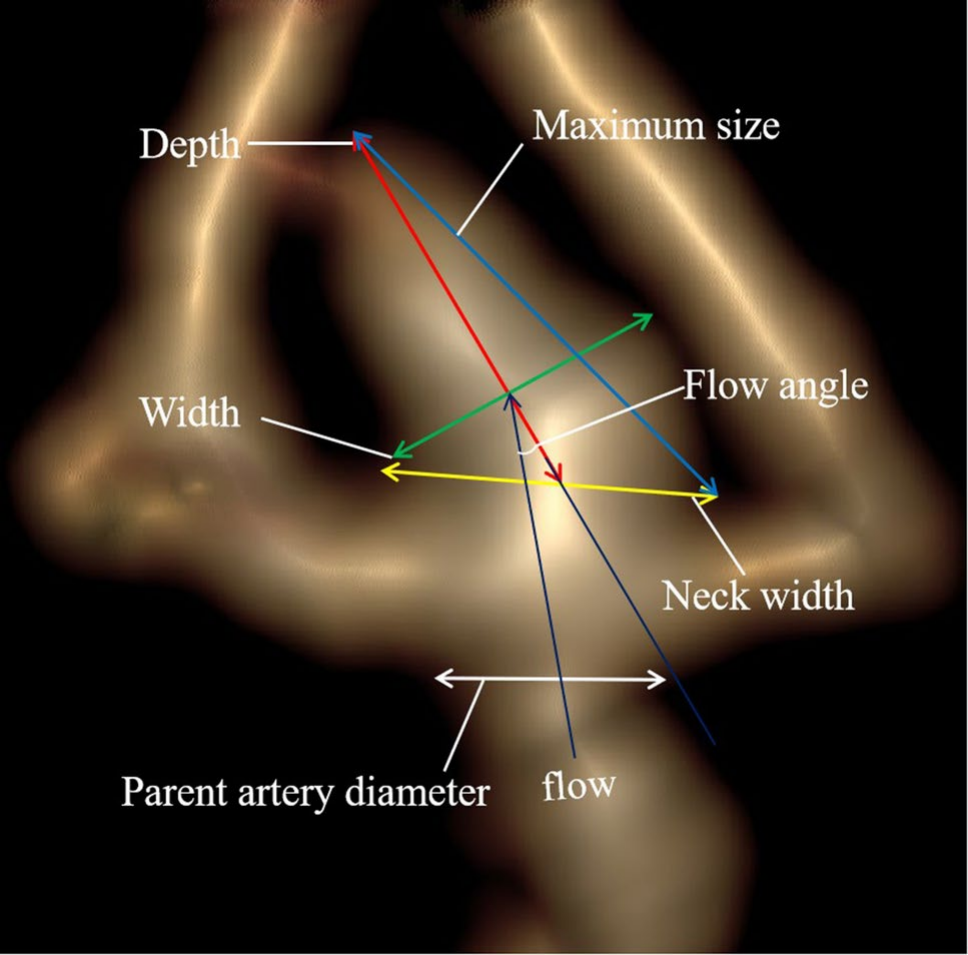
The image shows the method for the following dimension measurements: neck width (the largest cross-sectional diameter of the aneurysm neck), depth (the longest diameter between the neck and dome), width (the maximum distance vertical to depth), maximum size (the largest measurement in terms of maximum dome diameter or width), flow angle (angle between aneurysm depth vector and the vector of the centerline of the parent artery) and parent artery diameter (defined as the largest cross-sectional diameter of the artery)

The locations of the aneurysms were classified as the internal carotid artery (ICA), middle cerebral artery (MCA), anterior cerebral artery (ACA), anterior communicating artery (ACoA), posterior communicating artery (PCoA) or posterior circulation artery (PCA). The categorical morphological variables included the origin of the aneurysm (sidewall or bifurcation type), shape of the aneurysm (regular or irregular shape) and number of aneurysms. Aneurysms located at parent artery bifurcations were defined as the bifurcation type, and those originating from only one parent vessel were defined as the sidewall type [33]. An aneurysm with a lobular or daughter sac was defined as having an irregular shape. Multiple aneurysms were defined as the presence of more than 2 aneurysms in a single patient. The size of the aneurysm (maximum size, neck width, depth and width), the flow angle (FA) and the diameter of the parent artery were measured manually (Fig. 1). In addition, four secondary geometric morphology indices, including the aspect ratio (AR, depth/neck width), size ratio (SR, depth/parent artery diameter), depth-to-width ratio (DW, depth/width) and bottleneck factor (BF, width/neck width), were calculated. These variables have been clearly depicted in the previous literature [31].

### Splitting aneurysms for DL model development

**Fig. 2:**
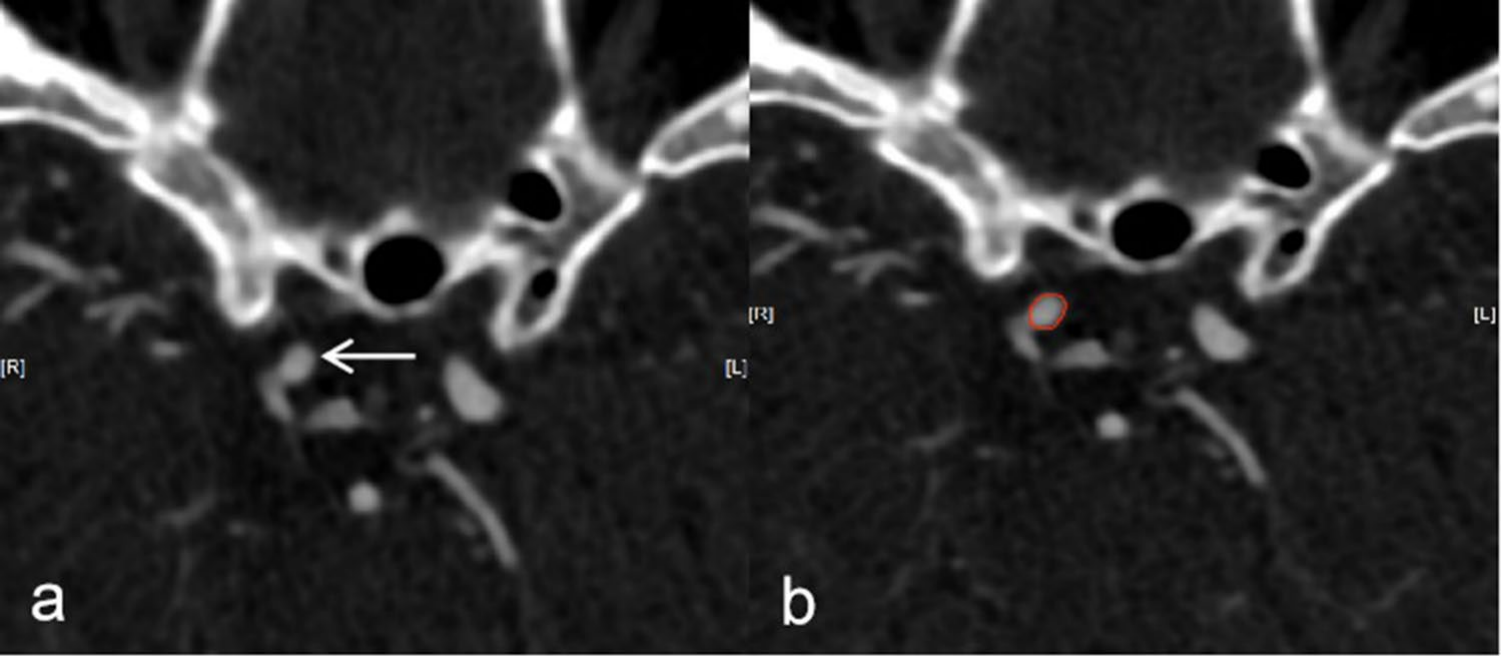
The image shows an aneurysm (**a**, arrow) and the region of interest of the aneurysm (**b**)

Two neuroradiologists used the Dr. Wise Multimodal Research Platform (https://keyan.Deepwise.com) to manually annotate the aneurysm contours layer-by-layer on the CTA images. First, the window width and level of the delineated CT image were adjusted to (400, 1000). The region of interest (ROI) of the aneurysm was subsequently positioned according to the aneurysm outline (Fig. 2). If there were any discrepancies, a third reader (with 25 years of experience in neuroradiology) joined the discussion to determine the final aneurysm outline. After aneurysm segmentation, all the images were randomly divided into training and internal validation sets with no duplication of data between the two sets.

The DL modeling process includes image preprocessing, feature extraction and classification. For image preprocessing, all images of the maximum slices of the aneurysm were resized to a fixed scale of 112 × 112 for two-dimensional (2D) modeling. Then, random horizontal flip, random vertical flip and random rotation by 30 degrees were applied to increase the amount of data and improve the utilization of the training set. For model training, a residual pretrained network (ResNet-34), which is a classic convolutional neural network (CNN) model, was used. In the process of training the model, we update the parameters. To match the number of classes, the output size was changed to 2.

During training, the batch size and the initial learning rate were set to 128 and 0.001, respectively. The number of epochs for model iteration was 500. The CrossEntropyLoss function was used to calculate the loss between the model’s prediction and the real class. We used the Adam optimization algorithm to update the neural network weights iteratively. We randomly selected the initial values of the weights from a uniform distribution, and the bias was initialized to 0. To output the classification predictions, the final DLM was chosen if the model had the highest accuracy values within 500 training epochs.

### Construction of different models

Six prediction models were constructed based on three types of features (clinical, morphological and DLM output probabilities) to determine the ability of these signatures to predict the stability of aneurysms. Model A (clinical features): Several basic clinical features of the patient, such as age, gender and alcohol consumption status, were used to construct Model A to assess the stability of the aneurysm via an LR ML algorithm. Model B (morphological features): The difference between Model B and Model A is that the input variables of Model B are related to the morphological features of the aneurysms, such as their location and the size. Model C (CTA images): This model, which is based on a convolutional neural network (ResNet-34), extracts DL features from the CTA images. Model D (clinical features and Model C’s prediction probability): Clinical features and the DLM output (prediction probability) of Model C are used as inputs to Model D, which is then built via an LR algorithm. Model E (clinical and morphological features): Clinical features and morphological features are used as inputs to Model E, which is then built via an LR algorithm. Model F (clinical features, morphological features and Model C’s prediction probability): This Model F uses an LR algorithm with clinical features, morphological features and Model C output probability as inputs.

### Statistical analysis

Statistical analyses were performed using SPSS software (version 25.0, IBM Corp., Armonk, NY, USA) and R software (version 4.2.2; www.r-project.org). Categorical features are presented as numbers (%) and were analyzed using the chi-square test or Fisher’s exact test. Continuous features are reported as the means ± standard deviations, and the Shapiro–Wilk test was used to determine normality, followed by Student’s *t*-test or the Mann–Whitney *U*-test. Clinical and morphological features were screened via univariate and multivariate LR analyses on the basis of the training dataset. LR algorithms were used for ML modeling to assess aneurysm stability. We assessed the performance and clinical utility of the models with receiver operating characteristic (ROC) curve, area under the curve (AUC) with 95% confidence interval (CI), accuracy, sensitivity, specificity, calibration curve and clinical decision curve analyses. The Delong test was used to compare the differences in ROC curves between the different methods. A *P* value < 0.05 was considered to indicate statistical significance.

## Results

A total of 1041 patients with 1227 aneurysms (672 unstable aneurysms and 555 stable aneurysms) were included in this study. The clinical characteristics of the patients and the morphological parameters of the aneurysms in the unstable and stable groups are listed in Table 1. The proportions of patients with heart disease, diabetes mellitus and cerebrovascular sclerosis were greater in the stable group than in the unstable group (*P* < 0.005), and the patients were older. All the morphological parameters were correlated with aneurysm stability.

**Table 1:** Characteristics of patients and intracranial aneurysms in the stable and unstable groups

Patient clinical information	Unstable (n = 672)	Stable (n = 555)	*P*
Female (%)	434 (64.6)	352 (63.4)	0.676
Age (years)	57.4 ± 12.34	60.4 ± 12.44	< 0.001
Hypertension (%)	285 (42.4)	267 (48.1)	0.05
Heart disease (%)	37 (5.5)	65 (11.7)	< 0.001
Diabetes mellitus (%)	31 (4.6)	54 (9.7)	0.001
Cerebrovascular sclerosis (%)	63 (9.4)	121 (21.8)	< 0.001
Alcohol consumption (%)	138 (20.5)	103 (18.6)	0.427
Smoking (%)	167 (24.9)	129 (23.2)	0.546
SAH history (%)	25 (3.7)	23 (4.1)	0.768
*Aneurysms parameters*			
Location (%)			< 0.001
ACoA	178 (26.5)	43 (7.7)	
ACA	37 (5.5)	19 (3.4)	
MCA	108 (16.1)	99 (17.8)	
PCoA	236 (35.1)	76 (13.7)	
ICA	77 (11.5)	308 (55.5)	
PCA	36 (5.4)	10 (1.8)	
Multiple aneurysms (%)	153 (22.8)	192 (34.6)	< 0.001
Bifurcation (%)	439 (65.3)	183 (33.0)	< 0.001
Irregular shape (%)	442 (65.8)	32 (5.8)	< 0.001
Daughter sac (%)	299 (44.5)	18 (3.2)	< 0.001
Neck width (mm)	5.01 ± 2.19	4.08 ± 1.13	< 0.001
Depth (mm)	7.14 ± 3.82	3.43 ± 1.25	< 0.001
Width (mm)	6.47 ± 4.05	3.65 ± 1.26	< 0.001
Maximum size (mm)	8.43 ± 4.23	4.47 ± 1.45	< 0.001
Parent artery diameter (mm)	3.33 ± 0.86	4.00 ± 0.92	< 0.001
AR	1.48 ± 0.62	0.85 ± 0.24	< 0.001
DW	1.19 ± 0.38	0.95 ± 0.22	< 0.001
BF	1.30 ± 0.52	0.90 ± 0.18	< 0.001
SR	2.25 ± 1.30	0.89 ± 0.34	< 0.001
FA	121.1 ± 25.27	102.6 ± 28.44	< 0.001

SAH, subarachnoid hemorrhage; ACoA, anterior communicating artery; ACA, anterior cerebral artery; MCA, middle cerebral artery; PCoA, posterior communicating artery; ICA, internal carotid artery; PCA, posterior circulation artery; AR, aspect ratio; DW, depth-to-width ratio; BF, bottleneck factor; SR, size ratio and FA, flow angle

**Fig. 3:**
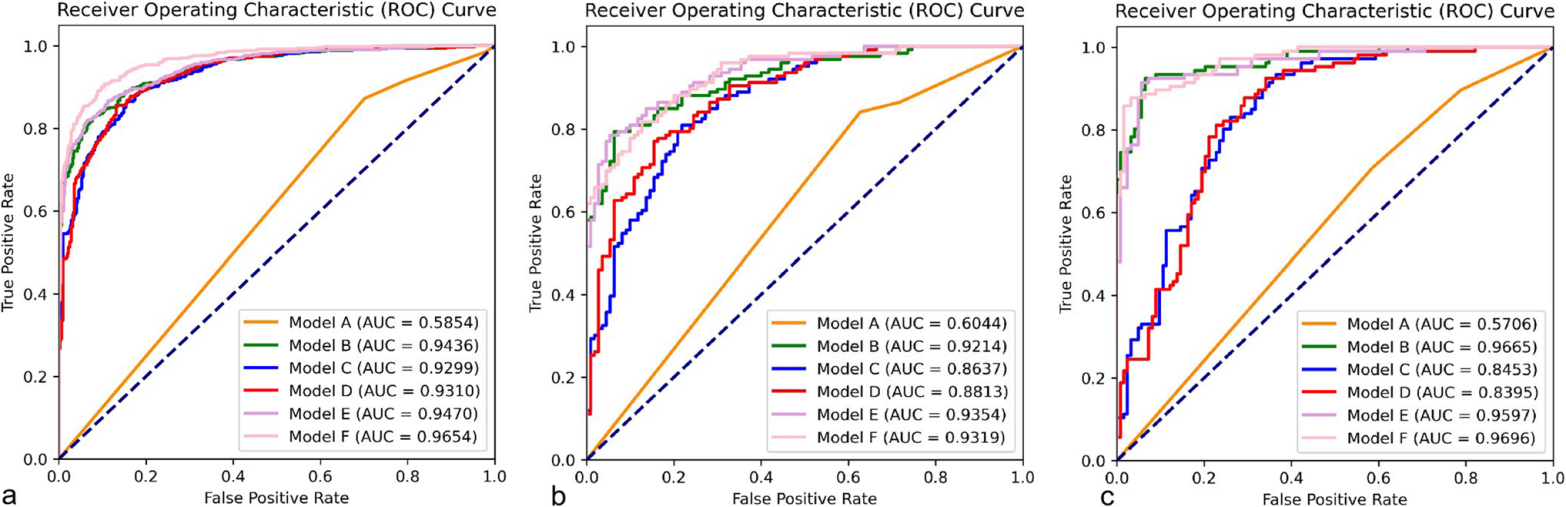
The ROC curves of the six prediction models. The AUCs of the six models in the training (**a**), internal validation (**b**) and external validation sets (**c**)

For the 1227 aneurysms, we randomly divided the dataset into training (833 patients with 547 unstable and 444 stable aneurysms) and internal validation (208 patients with 125 unstable and 111 stable aneurysms) sets (Table S1). Except for cerebrovascular sclerosis and multiple aneurysms, the other clinical and morphological characteristics were not significantly different between the training and internal validation sets. The clinical and morphological characteristics of the aneurysms in the training set are listed in Table 2. Multiple logistic regression revealed that heart disease, cerebrovascular sclerosis, multiple aneurysms, located at the bifurcation, irregular shape, maximum size and DW were associated with aneurysm stability. Six different models were subsequently constructed using these seven factors and the DLM output probability. The AUC, accuracy, sensitivity and specificity for the training set, internal validation set and external validation set are listed in Table 3 and Fig. 3. In the training set, Model A performed relatively poorly, with an AUC value of 0.585 (95% CI, 0.5589–0.6103). The AUCs of the other two prediction models (Model B and Model C) were 0.9436 (95% CI, 0.9309–0.9564) and 0.9299 (95% CI, 0.9138–0.9444), respectively, and Model B outperformed Model C. Model F (0.9654, 95% CI, 0.9560–0.9742) integrated three signatures and outperformed Model D (0.9310, 95% CI, 0.9153–0.9459) and Model E (0.9470, 95% CI, 0.9342–0.9598) (Fig. 3a).

**Table 2:** Characteristics of patients and intracranial aneurysms in the training set

Patient clinical information	Unstable (n = 547)	Stable (n = 444)	*P*	*P′*
Female (%)	356 (65.1)	287 (64.6)	0.9161	–
Age (years)	57.53 ± 12.26	60.12 ± 11.80	0.0006	0.2735
Hypertension (%)	232 (42.4)	209 (47.1)	0.1137	–
Heart disease (%)	32 (5.9)	51 (11.5)	0.0015	0.0150
Diabetes mellitus (%)	15 (2.7)	39 (8.8)	0.0075	0.0580
Cerebrovascular sclerosis (%)	46 (8.4)	90 (20.3)	< 0.0001	< 0.0001
Alcohol consumption (%)	113 (20.7)	86 (19.4)	0.5491	–
Smoking (%)	139 (25.4)	107 (24.1)	0.5822	–
SAH history (%)	20 (3.7)	20 (4.5)	0.4840	–
*Aneurysms parameters*				
Location (%)			< 0.0001	0.0920
ACoA	150 (27.4)	35 (7.9)		
ACA	28 (5.1)	13 (2.9)		
MCA	81 (14.8)	77 (17.3)		
PCoA	192 (35.1)	63 (14.2)		
ICA	66 (12.1)	249 (56.1)		
PCA	30 (5.5)	7 (1.6)		
Multiple aneurysms (%)	132 (24.1)	160 (36.0)	< 0.0001	0.0058
Bifurcation (%)	353 (64.5)	144 (32.4)	< 0.0001	< 0.0001
Irregular shape (%)	357 (65.3)	26 (5.9)	< 0.0001	< 0.0001
Daughter sac (%)	250 (65.1)	16 (3.6)	< 0.0001	0.8469
Neck width (mm)	5.02 ± 2.11	4.03 ± 1.07	< 0.0001	0.9550
Depth (mm)	7.19 ± 3.68	3.41 ± 1.22	< 0.0001	0.4341
Width (mm)	6.52 ± 3.83	3.60 ± 1.18	< 0.0001	0.4299
Maximum size (mm)	8.47 ± 4.05	4.43 ± 1.38	< 0.0001	0.0080
Parent artery diameter (mm)	3.34 ± 0.87	3.99 ± 0.91	< 0.0001	0.0904
AR	1.50 ± 0.64	0.85 ± 0.25	< 0.0001	0.8966
DW	1.19 ± 0.38	0.96 ± 0.23	< 0.0001	0.0150
BF	1.32 ± 0.53	0.89 ± 0.17	< 0.0001	0.3560
SR	2.26 ± 1.25	0.88 ± 0.34	< 0.0001	0.9093
FA	120.63 ± 25.18	102.83 ± 28.09	< 0.0001	0.1810

SAH, subarachnoid hemorrhage; ACoA, anterior communicating artery; ACA, anterior cerebral artery; MCA, middle cerebral artery; PCoA, posterior communicating artery; ICA, internal carotid artery; PCA, posterior circulation artery; AR, aspect ratio; DW, depth-to-width ratio; BF, bottleneck factor; SR, size ratio and FA, flow angle. *P*, value of univariate analysis and *P′*, value of multivariate analysis

**Table 3:** The AUC, diagnostic accuracy rate, sensitivity and specificity of the DLMs in identifying unstable aneurysms, and results of the Delong test between Model F and other models

Training set	AUC (95% CI)	Accuracy	Sensitivity	Specificity	Delong test
Model A	0.5854 (0.5589–0.6103)	0.6165	0.8725	0.2986	< 2.2e-16
Model B	0.9436 (0.9309–0.9564)	0.8607	0.8561	0.8665	2.941e-07
Model C	0.9299 (0.9138–0.9444)	0.8537	0.8925	0.8054	2.446e-11
Model D	0.9310 (0.9153–0.9459)	0.8547	0.8780	0.8258	1.92e-10
Model E	0.9470 (0.9342–0.9598)	0.8729	0.8707	0.8756	3.217e-06
Model F	0.9654 (0.9560–0.9742)	0.9021	0.9071	0.8959	–
*Internal validation set*					
Model A	0.6044 (0.5465–0.6614)	0.6229	0.8413	0.3727	< 2.2e-16
Model B	0.9214 (0.8910–0.9518)	0.8390	0.8333	0.8455	0.2181
Model C	0.8637 (0.8171–0.9075)	0.7924	0.8730	0.7000	1.081e-05
Model D	0.8813 (0.8377–0.9221)	0.7966	0.8651	0.7182	0.0003083
Model E	0.9354 (0.9052–0.9599)	0.8432	0.8492	0.8364	0.01172458
Model F	0.9319 (0.8997–0.9597)	0.8347	0.8968	0.7636	–
*External validation set*					
Model A	0.5706 (0.5069–0.6320)	0.5502	0.7075	0.4146	< 2.2e-16
Model B	0.9665 (0.9465–0.9836)	0.8952	0.8396	0.9431	0.731
Model C	0.8453 (0.7876–0.8932)	0.7686	0.9057	0.6504	2.981e-08
Model D	0.8395 (0.7869–0.8896)	0.7729	0.8774	0.6829	5.474e-09
Model E	0.9597 (0.9325–0.9814)	0.9039	0.8491	0.9512	0.02828649
Model F	0.9696 (0.9505–0.9852)	0.8865	0.8962	0.8780	–

AUC, area under the curve; CI, confidence interval; DLM, deep learning model. Model A, clinical parameters model; Model B, morphological parameters model; Model C, deep learning features model; Model D, clinical + deep learning features model; Model E, clinical + morphological parameters model and Model F, clinical + deep learning + morphological parameters model

The internal validation set was used to assess the generality and broad applicability of the six models (Table 3). The results revealed that Model B, Model E and Model F had outstanding classification ability (all AUCs > 0.90), and Model E (0.9354, 95% CI, 0.9052–0.9599) was slightly better than Model B (0.9214, 95% CI, 0.8910–0.9518) and Model F (0.9319, 95% CI, 0.8997–0.9597) (Fig. 3b).

A total of 197 patients with 229 IAs (106 unstable and 123 stable IAs) from Banan Hospital were included in the external validation set (Table S2). Cerebrovascular sclerosis and all the morphological characteristics of aneurysms are related to aneurysm stability. We further validated the six models using this set and found that Model B (0.9665, 95% CI, 0.9465–0.9836) performed better than Model C (0.8453, 95% CI, 0.7876–0.8932). Similarly, Model F (0.9696, 95% CI, 0.9505–0.9852) outperformed the other models (Table 3 and Fig. 3c), but the Delong test showed no significant difference between Model F and Model B (*P* = 0.731).

**Fig. 4:**
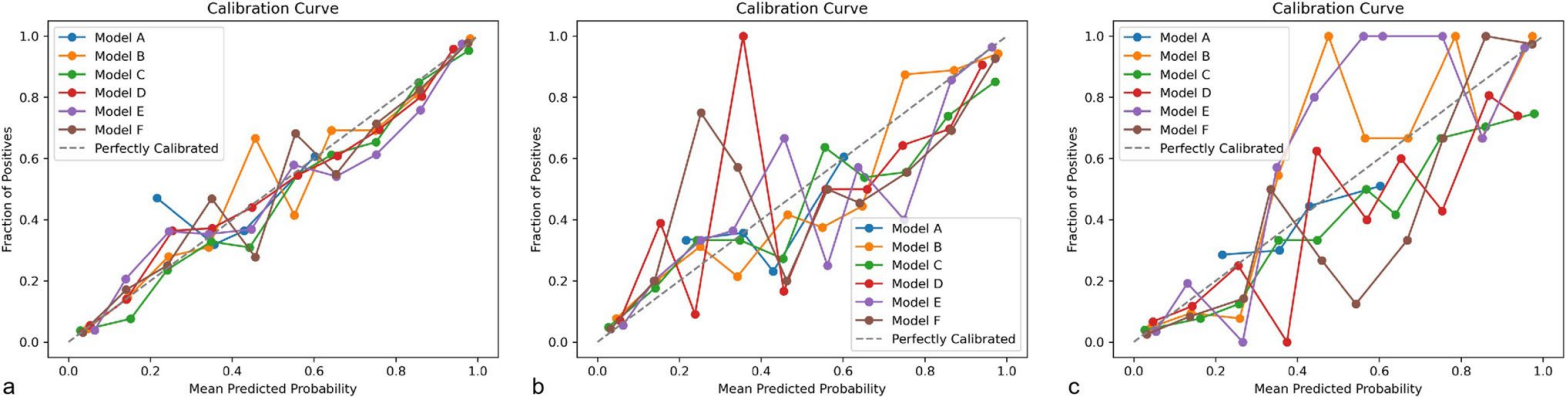
The predicted probabilities of the six models were evaluated through a calibration curve. **a**: training set; **b**: internal validation set and **c**: external validation set

**Fig. 5:**
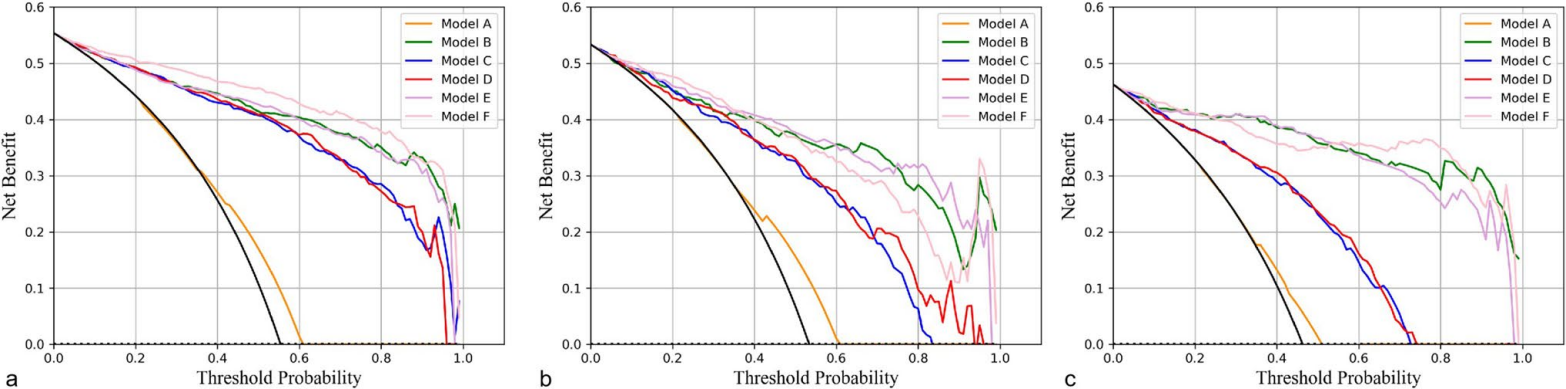
The practical clinical value of the six models was evaluated through clinical decision curve analysis. **a**: training set; **b**: internal validation set and **c**: external validation set

A calibration curve was drawn to observe whether the predicted probability of the classification model was close to the true probability. The calibration curve revealed that Model F had the best fit in both the training set, test set and external validation set (Fig. 4). The same phenomenon was observed in the decision curve (Fig. 5). The clinical decision curve of Model F exhibited high net benefit and excellent clinical applicability over a wide range of risk thresholds. Compared with model-free decision-making, full-model decision-making and model (A/B/C/D/E) decision-making, Model F significantly improves decision-making quality, and its curve shape is smooth and stable, confirming its effectiveness in actual clinical applications.

## Discussion

In this study, we developed six different models based on patient clinical characteristics, conventional morphological parameters and DL features of CTA images for the prediction of aneurysm stability; these models were validated with internal and external datasets. We found that Model F, which integrated the clinical characteristics, conventional morphological parameters and DL features of the CTA images, outperformed the other models and can be used for distinguishing aneurysm stability.

Assessing the stability of UIAs is still a complex matter due to the inconsistency of existing research. Several clinical factors, such as age, female sex, hypertension, cigarette smoking and excessive alcohol consumption, are associated with aneurysm stability [12, 32]. For example, according to the PHASES score, previous SAH due to RIA was identified as a risk factor for UIA rupture [13]. However, according to the ELAPSS score, previous SAH due to RIA was believed to have a negative effect on UIA growth [16]. According to the PHASES score and the ELAPSS score in this study, old age (> 70 years and > 60 years, respectively) was associated with an increased risk of UIA rupture and growth [13, 16]. However, another study revealed that younger patients are more likely to have RIAs [14]. In addition, an almost lifelong follow-up study of UIAs reported that age < 40 years, cigarette smoking and female sex were associated with UIA growth, and a new scoring system based on these three factors was significantly better than the ELAPSS score [18]. The scores have poor diagnostic accuracy and, therefore, require further improvement. Similarly, our results showed that Model A (clinical parameters) had poor predictive ability in the external validation set (AUC = 0.5706). This means that patients’ clinical factors alone are not sufficient to predict the stability of UIAs.

Morphological features are important markers for distinguishing the stability of UIAs. The size and location of the UIAs were used for treatment decision stratification [34]. Other factors, such as shape, AR, SR, DW, BF and FA, were also found to be related to aneurysm stability [13–16, 23, 31, 32]. In this study, a similar result was obtained and showed that the presence of multiple aneurysms, location at a bifurcation, irregular shape, maximum size and DW were the most important morphological features for distinguishing the stability of UIAs. Even if only five features were used, Model B (AUC = 0.9436) was able to distinguish the stability of UIA effectively. Furthermore, when clinical factors were added to the morphological features, the discrimination power of Model E (AUC = 0.9470) improved. These results further demonstrated the important role of morphological features in identifying UIA stability.

With the continuous development of AI, great progress has been made in aneurysm segmentation and automatic diagnosis. However, for distinguishing the stability of UIAs, the applications of AI are still in their infancy. In China, several AI models of aneurysm detection have been used in clinical practice, such as Deepwise & League of PHD technology and United Imaging Intelligence, which can detect aneurysms sensitively, but the risk of aneurysm rupture cannot be determined. ML and radiomics-based studies have been used to identify the risk for aneurysm rupture, but the results remain controversial. Liu et al. demonstrated that morphological features extracted from PyRadiomics can be used for assessing aneurysm stability and that flatness is the most important morphological determinant [26]. Some studies have shown that ML models perform better than conventional LR, and the PHASES scoring methods do and have the potential to use ML for aneurysm stability assessment [23, 27]. However, Calvin et al. reported that radiomics features extracted from angiography images had no additional value in terms of identifying aneurysm rupture risk [28]. Furthermore, a recent multicenter study conducted in China indicated that ML models did not outperform conventional LR in predicting the future rupture status of UIAs [25]. Therefore, we constructed a CNN-based DLM using CTA images to predict the stability of aneurysms. Our DL feature model (Model C) had an AUC of 0.8453 in the external validation set, and the accuracy, sensitivity and specificity were 0.7686, 0.9057 and 0.6504, respectively. When clinical factors were added to the DL features, the AUCs (0.9310 and 0.8813) of Model D improved in the training set and internal validation set, respectively. However, compared with that of Model C, the AUC (0.8395) of Model D decreased on the external validation set. Therefore, the results from the DL feature model are not stable.

The combination of features may be more useful for distinguishing the stability of aneurysms. Chen et al. reported that combining clinical, aneurysm morphological and hemodynamic features further improved discrimination performance [25]. Another study revealed that combining morphological and radiomic features further improved discrimination performance [35]. A recent study reported that combining clinical, morphological and radiomic features can improve the risk prediction of UIA rupture [36]. In our study, Model F, which integrates clinical, aneurysm morphological and DL parameters, showed the best performance in the external validation set (AUC = 0.9696). On the one hand, our results from the external validation set further increase the credibility of our model and indicate that DL methods have the potential to distinguish aneurysm stability. On the other hand, our results also further confirmed that the combination of features outperformed a single feature, but the Delong test showed no significant difference between Model F and Model B. A large multicenter study is still needed to further validate our results in the future.

This study has several strengths. First, we constructed six models and validated them with internal and external validation datasets based on a large sample. Second, DL features have been used and confirmed to play an important role in identifying unstable aneurysms. Third, our results showed that the DLM, which integrates the clinical, morphological and DL parameters of the aneurysm, outperformed the other models in predicting aneurysm stability.

### Limitations

There are several limitations in this study. First, this was a retrospective study, and some stable aneurysms may grow or rupture in the future. Therefore, long-term follow-up is needed. Second, the size and morphology of RIAs may change, which may generate a possible bias. Third, maximum slices of aneurysm images were used to construct 2D models, and some important factors that may affect aneurysm stability may have been missed, possibly leading to bias. More advanced DL algorithms are needed. Fourth, in this study, a CNN-based DLM was used to identify unstable aneurysms and was not compared with other DL algorithms; therefore, we are not sure which methods are better.

## Conclusions

This study describes the development of a CNN-based DLM using CTA images to assess aneurysm stability. The DLM, which integrates the clinical, morphological and DL parameters of the aneurysm, outperforms other models when assessing aneurysm stability and has the potential to aid in clinical decision-making to prevent the rupture of unstable UIAs and avoid unnecessary treatment of stable UIAs.

## Supplementary Information

Below is the link to the electronic supplementary material.

Supplementary file1 (DOCX 17 KB)

Supplementary file2 (DOCX 17 KB)
