# Supplementary material for: Assessment of the stability of intracranial aneurysms using a deep learning model based on computed tomography angiography
Source: Radiol Med. 2024 Dec 12;130(2):248–57. doi: 10.1007/s11547-024-01939-z (PMC11870988; doi:10.1007/s11547-024-01939-z)
Supplement: Supplementary file 1 — Supplementary file1 (DOCX 17 KB) [file 11547_2024_1939_MOESM1_ESM.docx]

| Table S1. Characteristics of patients and intracranial aneurysms in the training and internal validation set. | | | |
| --- | --- | --- | --- |
| Patient clinical information | Training set (n=991) | Internal validation set (n=236) | *P* |
| Female (%) | 643 (64.9) | 143 (60.6) | 0.228 |
| Age (Years) | 58.69 ± 12.12 | 59.16 ± 13.85 | 0.636 |
| Hypertension (%) | 441 (44.5) | 111 (47.0) | 0.511 |
| Heart disease (%) | 83 (8.4) | 19 (8.1) | >0.99 |
| Diabetes mellitus (%) | 64 (6.5) | 21 (8.9) | 0.199 |
| Cerebrovascular sclerosis (%) | 136 (13.7) | 48 (20.3) | 0.015 |
| Alcohol consumption (%) | 199 (20.1) | 42 (17.8) | 0.466 |
| Smoking (%) | 246 (24.8) | 50 (21.2) | 0.271 |
| SAH history (%) | 40 (4.0) | 8 (3.4) | 0.852 |
| Aneurysms parameters |  |  |  |
| Location (%) |  |  | 0.271 |
| ACoA | 185 (18.7) | 36 (15.3) |  |
| ACA | 41 (4.1) | 15 (6.4) |  |
| MCA | 158 (15.9) | 49 (20.8) |  |
| PCoA | 255 (25.7) | 57 (24.2) |  |
| ICA | 315 (31.8) | 70 (29.7) |  |
| PCA | 37 (3.7) | 9 (3.8) |  |
| Multiple aneurysms (%) | 292 (29.5) | 53 (22.5) | 0.036 |
| Bifurcation (%) | 497 (50.2) | 125 (53.0) | 0.469 |
| Irregular shape (%) | 383 (38.6) | 91 (38.6) | >0.99 |
| Daughter sac (%) | 266 (26.8) | 51 (21.6) | 0.116 |
| Neck width (mm) | 4.58 ± 1.79 | 4.64 ± 2.08 | 0.657 |
| Depth (mm) | 5.49 ± 3.42 | 5.32 ± 3.75 | 0.494 |
| Width (mm) | 5.21 ± 3.29 | 5.13 ± 3.90 | 0.728 |
| Maximum size (mm) | 6.66 ± 3.74 | 6.54 ± 4.19 | 0.651 |
| Parent artery diameter (mm) | 3.64 ± 0.94 | 3.64 ± 0.96 | 0.939 |
| AR | 1.21 ± 0.59 | 1.14 ± 0.50 | 0.121 |
| DW | 1.09 ± 0.34 | 1.07 ± 0.33 | 0.636 |
| BF | 1.13 ± 0.46 | 1.09 ± 0.39 | 0.144 |
| SR | 1.64 ± 1.17 | 1.58 ± 1.32 | 0.491 |
| FA | 112.66 ± 27.95 | 113.10 ± 29.74 | 0.831 |
| SAH, subarachnoid hemorrhage; ACoA, anterior communicating artery; ACA, anterior cerebral artery; MCA, middle cerebral artery; PCoA, posterior communicating artery; ICA, internal carotid artery; PCA, posterior circulation artery; AR, aspect ratio; DW, depth-to-width ratio; BF, bottleneck factor; SR, size ratio; FA, flow angle. | | | |
