# Supplementary material for: Assessment of the stability of intracranial aneurysms using a deep learning model based on computed tomography angiography
Source: Radiol Med. 2024 Dec 12;130(2):248–57. doi: 10.1007/s11547-024-01939-z (PMC11870988; doi:10.1007/s11547-024-01939-z)
Supplement: Supplementary file 2 — Supplementary file2 (DOCX 17 KB) [file 11547_2024_1939_MOESM2_ESM.docx]

| Table S2. Clinical information and manual parameters of the aneurysms in the external validation set. | | | |
| --- | --- | --- | --- |
| Patient clinical information | Unstable (n=106) | Stable (n=123) | *P* |
| Female (%) | 72 (67.9) | 81 (65.9) | 0.848 |
| Age (Years) | 64.41 ± 12.43 | 67.03 ± 11.53 | 0.099 |
| Hypertension (%) | 37 (34.9) | 41 (33.3) | 0.912 |
| Heart disease (%) | 22 (20.8) | 30 (24.4) | 0.619 |
| Diabetes mellitus (%) | 63 (59.4) | 63 (51.2) | 0.266 |
| Cerebral vascular sclerosis (%) | 11 (10.4) | 26 (21.1) | 0.043 |
| Alcohol consumption (%) | 17 (16.0) | 32 (26.0) | 0.094 |
| Smoking (%) | 22 (20.8) | 32 (26.0) | 0.436 |
| SAH history (%) | 1 (0.9) | 0 (0.0) | 0.941 |
| Aneurysms parameters |  |  |  |
| Location (%) |  |  | <0.001 |
| ACoA | 43 (40.6) | 10 (8.1) |  |
| ACA | 2 (1.9) | 3 (2.4) |  |
| MCA | 15 (14.2) | 20 (16.3) |  |
| PCoA | 36 (34.0) | 28 (22.8) |  |
| ICA | 5 (4.7) | 57 (46.3) |  |
| PCAs | 5 (4.7) | 5 (4.1) |  |
| Multiple aneurysms (%) | 27 (25.5) | 27 (22.0) | 0.639 |
| Bifurcation (%) | 82 (77.4) | 34 (27.6) | <0.001 |
| Irregular shape (%) | 79 (74.5) | 5 (4.1) | <0.001 |
| Daughter sac (%) | 68 (64.2) | 3 (2.4) | <0.001 |
| Neck width (mm) | 5.05 ± 1.71 | 3.67 ± 1.01 | <0.001 |
| Height (mm) | 5.48 ± 2.70 | 2.46 ± 0.96 | <0.001 |
| Depth (mm) | 5.87 ± 2.79 | 2.60 ±1.04 | <0.001 |
| Width (mm) | 6.34 ± 3.54 | 3.16 ± 1.03 | <0.001 |
| Maximum size (mm) | 7.71 ± 3.34 | 3.90 ± 1.22 | <0.001 |
| Parent artery diameter (mm) | 3.41 ± 0.98 | 3.86 ± 0.94 | 0.001 |
| AR | 1.21 ± 0.50 | 0.73 ± 0.28 | <0.001 |
| DW | 1.00 ± 0.34 | 0.83 ± 0.24 | <0.001 |
| BF | 1.26 ± 0.51 | 0.86 ± 0.17 | <0.001 |
| SR | 2.05 ± 1.21 | 0.74 ± 0.33 | <0.001 |
| FA (°) | 133.24 ± 23.04 | 109.16 ± 28.81 | <0.001 |
| SAH, subarachnoid hemorrhage; ICA, internal carotid artery; MCA, middle cerebral artery; ACA, anterior cerebral artery; ACoA, anterior communicating artery; PCoA, posterior communicating artery; PCA, posterior circulation artery; AR, aspect ratio; SR, size ratio; DW, depth-to-width ratio; BF, bottleneck factor; FA, flow angle. | | | |
